# A Tale of Two “Unexpected” Asystoles

**DOI:** 10.3390/jcdd12070257

**Published:** 2025-07-04

**Authors:** Giacomo Mugnai, Bruna Bolzan, Elena Franchi, Luca Tomasi

**Affiliations:** Division of Cardiology, Cardio-Thoracic Department, University Hospital of Verona, 37126 Verona, Italy

**Keywords:** asystole, defibrillator, life vest

## Abstract

We report two cases of prolonged “unexpected” asystoles in patients with a wearable cardioverter-defibrillator (WCD) and a subcutaneous implantable cardioverter-defibrillator (ICD), respectively, which were promptly recognized and successfully managed. As these devices are designed to recognize and treat malignant tachyarrhythmias but do not provide pacing capabilities, it is crucial to identify patients with paroxysmal conduction disorders who might require backup pacing. For this reason, it is also important to leverage the monitoring features of both devices and their ability to detect the occurrence of bradyarrhythmias.

## 1. Introduction

Currently, given the increased incidence of life-threatening ventricular arrhythmias in patients with cardiomyopathy and low ejection fraction, a wearable cardioverter-defibrillator (WCD; Zoll LifeVest) is generally recommended for patients who are not immediate candidates for implantable cardioverter defibrillators (ICDs) [1]. According to current guidelines [2], the WCD should be considered for adult patients with a secondary prevention ICD indication who are temporarily not candidates for ICD implantation (Class IIa, Level of Evidence C).

However, such patients often have structural heart diseases that also present a high incidence of conduction disorders; additionally, these disorders may be worsened by the concomitant use of antiarrhythmic drugs.

Although WCDs do not offer anti-bradycardia treatment, they can be helpful by alerting bystanders with an audible tone, suggesting that they call for help and perform cardiopulmonary resuscitation. Unlike endocavitary ICDs, which are primarily designed to recognize and treat malignant ventricular tachycardias but can also offer pacing support in cases of bradyarrhythmias (functioning as pacemakers), subcutaneous ICDs are able to detect and treat ventricular tachyarrhythmias but do not allow the direct recording of bradyarrhythmias [3,4]. According to the guidelines [2], subcutaneous ICDs should be considered as an alternative to transvenous ICDs in patients with an ICD indication when pacing therapy for bradycardia, cardiac resynchronization, or ATP is not needed (Class IIa, Level of Evidence B).

Liang et al. [1] reported that asystoles and severe bradycardias were found in 0.5% of patients with WCD. Similarly, a large pooled analysis of nearly 400 patients with a subcutaneous ICD found the rate of bradycardia-associated complications to be 0.3% [5].

Herein, we describe two cases of prolonged, “unexpected” asystoles in patients with a WCD and an S-ICD, respectively, which were promptly recognized and successfully treated.

## 2. Case Presentation

Case #1: A 77-year-old male with ischemic dilated cardiomyopathy associated with severely depressed left ventricular ejection fraction and previous aortocoronary bypass presented to our outpatient clinic for a routine check of his subcutaneous ICD (Emblem, Boston Scientific, USA). A few years prior, the patient had undergone a bioprosthetic mitral valve replacement, which was subsequently complicated by endocarditis and septic cerebral embolization, necessitating a redo mitral valve replacement.

Subcutaneous ICD interrogation did not reveal any major arrhythmias, but the SMART Pass (high-pass filter) had deactivated. The SMART Pass is a 9 Hz high-pass filter developed to avoid inappropriate shocks due to T-wave oversensing. This algorithm functions by reducing T-wave amplitude without affecting the sensing of the QRS complexes, thereby improving the QRS-to-T wave ratio. While this algorithm was developed to avoid undersensing of low-amplitude ventricular arrhythmias, the SMART Pass is specifically designed to auto-deactivate in the presence of low-amplitude signals (0.25 mV).

**Figure 1:**
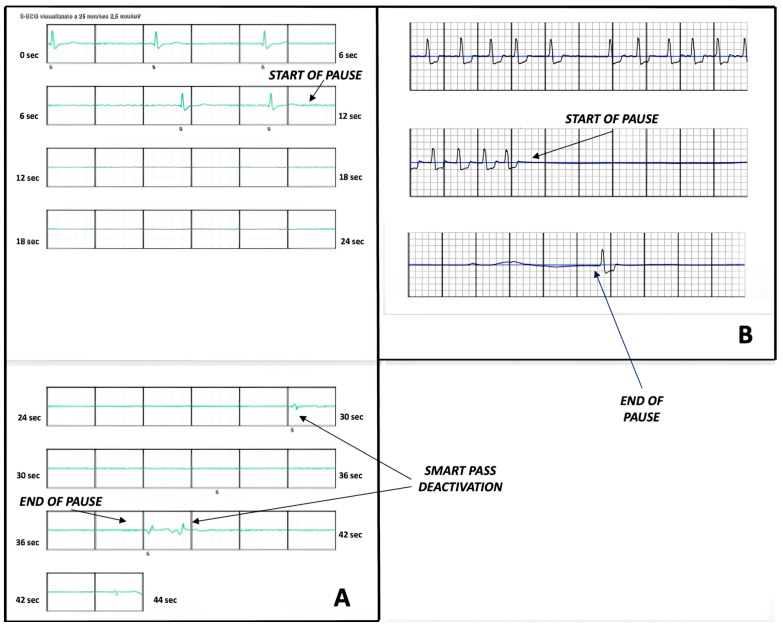
(**A**) The tracing from subcutaneous ICD shows a marked bradycardia with a prolonged asystolic pause of 18 s. (**B**) The tracing from LifeVest reveals an episode of asystole of 12 s.

Upon reviewing the SMART Pass deactivation episode, we found a prolonged, marked bradycardia lasting 44 s, with a maximum asystolic pause of 18 s (Figure 1A). The episode was recorded the week prior, in the afternoon at 2:13 p.m., after lunch and while the patient was resting on the couch. Surprisingly, the episode caused only mild dizziness. After device interrogation, the patient was immediately admitted to our Cardiology service for ECG monitoring. His medications included amiodarone 200 mg per day and bisoprolol 5 mg per day. The following day, he was implanted with a leadless pacemaker.

Case #2: A 76-year-old male with valvular cardiomyopathy, permanent atrial fibrillation, and a history of mitral valve replacement followed by a redo surgical procedure due to endocarditis had recently been admitted for recurrent heart failure. Severe left ventricular dysfunction was identified during the hospital stay, leading to the patient receiving a WCD (Zoll LifeVest) with a plan to reassess left ventricular ejection fraction thereafter. Two months later, the patient was awakened by the device’s acoustic alarm at 5:00 a.m. and presented to the Emergency Department. Our review revealed a prolonged pause of 12 s (Figure 1B). Therefore, the patient was admitted to Cardiology for continuous ECG monitoring. He was receiving medical therapy with bisoprolol 2.5 mg per day.

As the patient required both pacing and defibrillation therapy and the implantation of an endocavitary device was deemed high-risk for infections, we opted for a surgical procedure via a mini-thoracotomy approach, combining epicardial lead implantation in the right and left ventricles with the concurrent placement of a subcutaneous ICD (Emblem, Boston Scientific, Marlborough, MA, USA). The procedure was successfully performed, and the patient was discharged a few days later.

## 3. Discussion

Although asystole episodes are infrequent in patients with a WCD and an S-ICD, the presence of cardiomyopathies, ischemic heart disease, and concomitant antiarrhythmic therapies can nonetheless result in significant bradyarrhythmias [1]. Over three-quarters of these asystole episodes resulted in unconsciousness, hospitalization, or death, with survival rates after the episodes being approximately 44% [1]. As in our cases, the WCD can be extremely helpful in patients with severe bradyarrhythmias by alerting bystanders to notify emergency medical services and to perform early cardiopulmonary resuscitation, as well as by detecting episodes that warrant appropriate permanent device implantation.

On the other hand, S-ICDs do not commonly allow the detection of bradyarrhythmias. In fact, the criteria currently adopted for SMART Pass deactivation may be insufficient for the diagnosis of many forms of bradyarrhythmias [3,4]. Of note, the novel extravascular ICD (Aurora EV-ICD, Medtronic, MN, USA), recently released on the market, will be able to offer pause prevention pacing (backup bradycardia pacing), which can be lifesaving in cases of asystole or marked bradyarrhythmias. However, larger clinical and real-world data are still needed to confirm its safety and efficacy in such patients.

Both patients had no history of bradyarrhythmias, and no suspicion of bradycardias was raised before ICD implantation. Telemetry during the hospital stay did not record any bradyarrhythmic events. In general, one might hypothesize that certain ECG baseline parameters could help identify patients prone to bradyarrhythmic events. Pauses, first- and second-degree AV blocks, and bifascicular or left bundle branch blocks might be potential markers that should prompt consideration for pacing in such patients. However, neither of our patients exhibited the aforementioned ECG markers. During follow-up, both patients showed a percentage of pacing less than 0.1%, confirming that the conduction disorder was truly paroxysmal.

In conclusion, although severe bradycardias are usually unexpected, their incidence in patients with a WCD and an S-ICD is not negligible. While these devices are not designed to recognize and treat asystoles and bradyarrhythmias, it is extremely important, and sometimes lifesaving, to detect and manage them as soon as possible.

## Data Availability

The data presented in this study are available on request from the corresponding author.
